# CT-based immune radiomic signature for prognosis and prediction of immunotherapy and anticancer drug response in NSCLC

**DOI:** 10.3389/fimmu.2026.1767389

**Published:** 2026-04-29

**Authors:** Jianying Ma, Kaixing Guo, Yanpo Gao, Tengfei Li, Fei Li, Chaoqi Pang, Yunping Geng, Jiawei Guo

**Affiliations:** 1 Department of CT Imaging, Nanyang Central Hospital, Nanyang, China; 2 Department of Pharmacy, Affiliated Hospital of Hebei University of Engineering, Handan, China; 3 Department of Medicine and Health, Handan Vocational College of Science and Technology, Handan, China; 4 Department of Pharmacology, School of Medicine, Yangtze University, Jingzhou, China

**Keywords:** CT, drug response, immunotherapy, non-small cell lung cancer, prognosis, radiomics

## Abstract

**Background:**

Non-small cell lung cancer (NSCLC) accounts for more than 85% of lung cancers and remains the leading cause of cancer-related mortality worldwide, with dismal prognosis and pronounced inter-patient heterogeneity in responses to immune checkpoint inhibitors, chemotherapy and targeted therapies. Current approaches to characterising the tumour microenvironment (TME) immune landscape rely on invasive tissue biopsies, which are constrained by sampling bias and an inability to capture spatial and temporal heterogeneity. CT-based radiomics, which derives high-dimensional quantitative features from routine imaging, offers a non-invasive alternative; however, most existing radiomic signatures lack robust biological grounding and do not provide integrated prediction of multiple treatment responses.

**Objective:**

To develop and validate a non-invasive, immune-informed CT-based radiomic signature (CT-RadScore) that reflects TME immune phenotypes and to evaluate its utility for prognostic stratification and prediction of responses to immunotherapy and anticancer drugs in NSCLC.

**Methods:**

We analysed multiple NSCLC cohorts, including TCIA NSCLC Radiogenomics, TCGA LUAD+LUSC and an in-house cohort from Nanyang Central Hospital. Consensus clustering of immune profiles was used to derive immune subtypes, and weighted gene co-expression network analysis (WGCNA) linked immune infiltration patterns to gene modules. CT radiomic features were extracted using PyRadiomics, and machine-learning survival models (random survival forest [RSF], LASSO–Cox, Elastic Net–Cox) were compared to construct CT-RadScore. Functional validation included Tumour Immune Dysfunction and Exclusion (TIDE) analysis, Tracking Tumour Immunophenotype (TIP) profiling, in silico drug sensitivity prediction, single-cell RNA sequencing (scRNA-seq) and GPX2 knockdown experiments.

**Results:**

Consensus clustering identified two reproducible immune subtypes (immune-hot and immune-cold), which were validated across seven independent immune deconvolution algorithms. WGCNA revealed an immune-related gene module enriched for pathways governing innate and adaptive immunity. An RSF-integrated Cox model (COX+RSF) yielded CT-RadScore, composed of 12 non-redundant radiomic features, which demonstrated robust and externally validated prognostic performance (C-index: 0.791 in TCIA, 0.729 in TCGA and 0.844 in the in-house cohort). High CT-RadScore was associated with significantly worse overall survival (all log-rank P<0.0017) and remained an independent prognostic factor after adjustment for clinicopathological covariates. Low CT-RadScore predicted improved immunotherapy response (response rates 54.7–75% versus 30–34.4% in high-score groups; in-house AUC = 0.817) and was associated with lower TIDE scores. CT-RadScore further correlated with predicted sensitivity to key agents (for example, paclitaxel, gefitinib and carboplatin) and was mechanistically linked to GPX2-driven DNA replication licensing programmes and impaired antitumour immune cycling.

**Conclusion:**

CT-RadScore is a non-invasive, biologically anchored radiomic signature that captures TME immune phenotypes and tumour proliferative programmes. It provides independent prognostic information and robust prediction of immunotherapy and anticancer drug responses, with potential to refine risk stratification and guide personalised treatment selection in NSCLC. Prospective multicentre validation and methodological standardisation will be essential for clinical translation.

## 1 Introduction

Non-small cell lung cancer (NSCLC) accounts for more than 85% of all lung cancer cases and remains the leading cause of cancer-related mortality worldwide, with a 5-year survival of only about 15.9% amongst patients with advanced-stage disease (1). Despite major advances in systemic therapies for advanced NSCLC—including immune checkpoint inhibitors (ICIs) targeting PD-1/PD-L1 and CTLA-4, as well as chemotherapeutic and targeted molecular agents—clinical outcomes remain unsatisfactory: overall survival for most patients is still poor, and therapeutic responses are highly heterogeneous across individuals (2). These persistently poor survival outcomes, together with the marked interpatient heterogeneity in therapeutic response, highlight an urgent need for robust biomarkers with prognostic and predictive value, enabling refined risk stratification and more precise selection of immunotherapy, chemotherapy and targeted therapies to maximise individual patient benefit.

The tumour microenvironment (TME) plays a crucial role in both the progression of NSCLC and its response to treatment (3). Tumours with an “immune-hot” microenvironment, characterised by abundant tumour-infiltrating lymphocytes, including cytotoxic CD8^+^ T cells together with macrophages and dendritic cells, are more likely to benefit from immune checkpoint blockade (ICB), whereas “immune-cold” tumours, defined by a very low density of tumour-infiltrating lymphocytes and an enrichment of immunosuppressive populations such as regulatory T cells, myeloid-derived suppressor cells and M2-polarised tumour-associated macrophages, are generally associated with resistance to ICIs (4). Beyond shaping responses to ICIs, the immune compartment of the tumour microenvironment also critically modulates the efficacy of cytotoxic chemotherapy and molecularly targeted agents in NSCLC, as an immunosuppressive, chronically inflamed immune milieu can blunt treatment-induced tumour cell death, promote the survival of drug-tolerant clones and foster both *de novo* and acquired resistance, whereas therapeutic strategies that reprogram this immune contexture have been shown to partially restore chemosensitivity and enhance the activity of targeted therapies (5, 6). Current methods for assessing the immune landscape within the TME, such as immunohistochemistry for PD-L1 or multiplex immunofluorescence, rely heavily on invasive tissue biopsies (7). These techniques are limited by sampling biases, insufficient tissue quantity, and an inability to capture spatial or temporal heterogeneity within the TME (8, 9).

In this context, radiomics, a field that extracts high-dimensional quantitative features from medical imaging, particularly from computed tomography (CT), offers a promising solution. CT imaging is widely used in the diagnosis and staging of NSCLC, capturing spatial information that indirectly reflects TME characteristics like fibrosis, vascularisation, and immune cell infiltration (10–14). However, although an increasing number of radiomic studies have begun to correlate imaging features with diverse aspects of tumour biology, most investigations remain retrospective and predominantly correlative, with limited biological validation and only modest exploration of immune-related pathways or endpoints, which constrains their biological interpretability and translational impact (15). Most existing models remain limited by incomplete biological grounding and a lack of integration with immune-related treatment endpoints, particularly those across immunotherapy, chemotherapy and targeted agents, which further constrains their utility in guiding personalised therapeutic decisions. Thus, despite growing interest, current radiomic approaches still fall short of providing biologically grounded, immune-informed biomarkers for truly personalised cancer therapy.

Against this background, we aimed to develop and validate a non-invasive, immune-informed CT-based radiomic signature (CT-RadScore) that reflects the immune phenotype of the TME in NSCLC. Using immune-related gene expression programmes as a biological reference, we identified CT features significantly associated with immune functional states and integrated them into a composite radiomic score. We then assessed whether CT-RadScore could provide prognostic stratification and predict treatment response across independent patient cohorts. By serving as an imaging-based surrogate for the immune landscape of the TME, CT-RadScore has the potential to support more precise patient stratification and inform personalised therapeutic decision-making in NSCLC.

## 2 Result

### 2.1 Development and validation of immune infiltration consensus clusters

**Figure 1:**
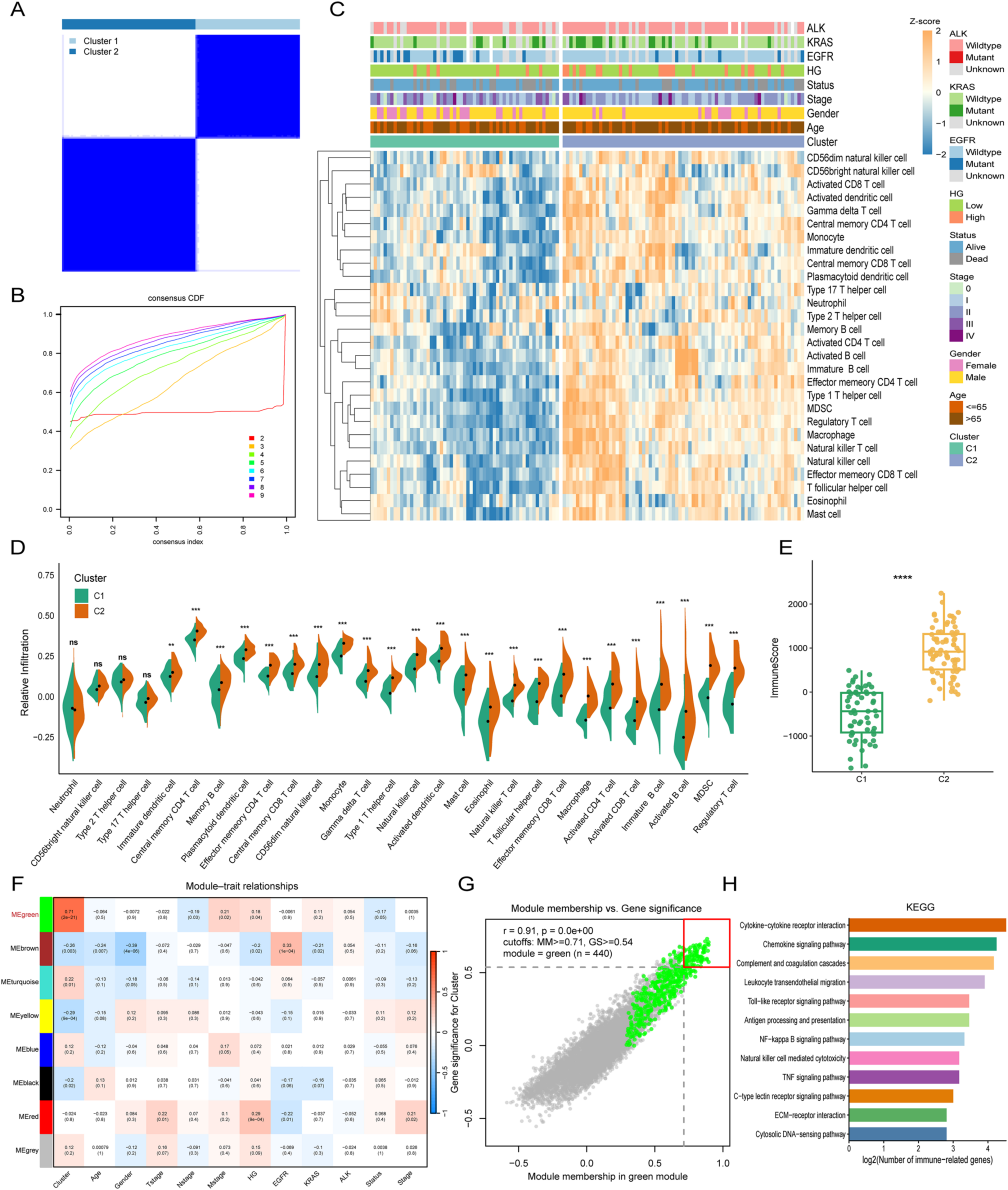
Identification of immune-related genes using two algorithms. **(A)** Consensus score matrix of all samples at k = 2. A higher consensus score between two samples indicates that they are more likely to be assigned to the same cluster across different iterations. **(B)** Cumulative distribution function (CDF) curves of the consensus matrix for each k (indicated by different colours). **(C)** Infiltration abundance of 28 immune cell subsets in the two clusters, evaluated by ssGSEA. **(D)** Distribution of infiltration levels of the 28 immune cell subsets between the two clusters. **(E)** Distribution of immune scores inferred by the ESTIMATE algorithm between the two clusters in the TCIA NSCLC Radiogenomics cohort (n = 129). **(F)** Correlation analysis between module eigengenes and clinical traits. **(G)** Strong correlation between gene significance (GS) and module membership (MM) in the green module (P = 0). Dots within the red rectangle were defined as immune-related genes with both high GS and MM. **(H)** Identification of 440 genes significantly associated with immune-related pathways. ** p < 0.01; *** p < 0.001; **** p < 0.0001.

Using ssGSEA to quantify the infiltration of 28 immune cell types (16), we performed consensus clustering (17) and ultimately partitioned the NSCLC samples into k (k = 2-9) clusters. The cumulative distribution function (CDF) curves of the consensus score matrix and the proportion of ambiguous clustering (PAC) statistic (18) indicated that k = 2 yielded the optimal number of clusters (**Figures 1A, B**; **Supplementary Figure 2A**). Consistently, the NbClust assessment (**Supplementary Figure 2B**) supported k = 2 as the most appropriate solution.

These two consensus clusters (C1 and C2) exhibited pronounced differences in immune infiltration, with C2 displaying a markedly higher overall abundance of infiltrating immune cells than C1 (**Figures 1C, D**). Accordingly, we defined C1 as an “immune-cold” tumour subtype and C2 as an “immune-hot” tumour subtype. To ensure that these consensus clusters were not artefacts driven by a specific analytical algorithm, we further validated the stability and robustness of the ssGSEA-based results using seven additional immune analysis methods, including TIMER, QUANTISEQ, MCPCOUNTER, xCell, EPIC, CIBERSORT and ESTIMATE (**Figure 1E**; **Supplementary Figure 2C**).

### 2.2 Identification of gene modules derived from immune infiltration patterns

During the WGCNA, the β was set to 10 (scale-free topology fit index R² = 0.863), which provided an appropriate power for network construction (**Supplementary Figure 2D**). Subsequently, eight modules were identified and distinguished by different colours. The module eigengene (i.e., the first principal component of gene expression within a given module) was taken as the representative of each module. The eigengene adjacency heatmap illustrated the relationships amongst module eigengenes (**Supplementary Figure 2E**). We then assessed the correlations between module eigengenes and multiple clinical traits, including immune clusters, age, sex, T stage, N stage, M stage, tumour histologic grade, EGFR/KRAS/ALK mutation status, survival status, and AJCC stage. The strongest module–trait association was observed between the green module and the immune clusters (**Figure 1F**). Within the green module, the correlation coefficient between gene significance (GS) and module membership (MM) reached 0.91, indicating a high-quality module definition (**Figure 1G**). To identify hub genes associated with immune infiltration patterns within the green module, we defined 440 genes with GS > 0.54 and MM > 0.71 as immune-related hub genes (**Figure 1G**).

### 2.3 KEGG pathway enrichment of the green module highlights immune-activating signalling cascades

To elucidate the biological functions represented by the green module, we performed KEGG pathway enrichment analysis based on the genes in this module. The top enriched pathways were almost exclusively immune-related, with prominent terms including “Cytokine–cytokine receptor interaction”, “Chemokine signalling pathway” and “Complement and coagulation cascades”, indicating a strong involvement in cytokine networks and inflammatory responses. Additional significantly enriched pathways, such as “Leukocyte transendothelial migration”, “Natural killer cell mediated cytotoxicity”, “Antigen processing and presentation”, “Toll-like receptor signalling pathway”, “NF-κappa B signalling pathway” and “TNF signalling pathway”, underscored a central role in both innate and adaptive antitumour immunity. We also observed enrichment of pattern-recognition and microenvironmental pathways, including the “C-type lectin receptor signalling pathway”, “Cytosolic DNA-sensing pathway” and “ECM–receptor interaction”, suggesting that genes in the green module participate in orchestrating immune cell recruitment, activation and effector functions within the tumour microenvironment (**Figure 1H**).

### 2.4 Development of an immune-related CT-based radiomics signature

**Figure 2:**
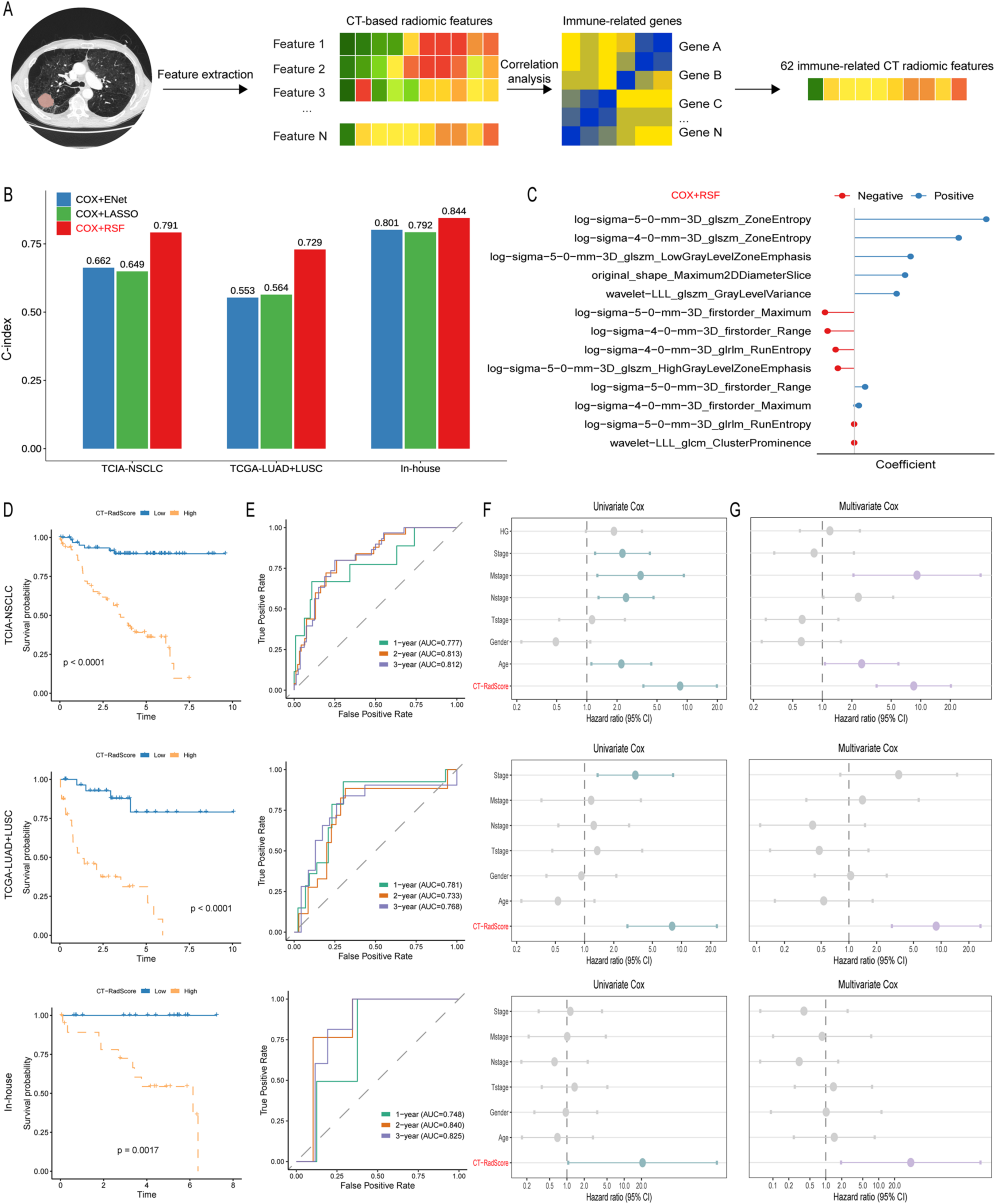
Development and validation of an immune-associated CT radiomics signature (CT-RadScore). **(A)** Schematic workflow for identifying immune-related radiomic features. Radiomic features were extracted from CT images and correlated with immune-related genes, yielding 62 radiomic features significantly associated with immune signatures. **(B)** Performance comparison of three modelling strategies (Cox+Elastic Net, Cox+LASSO, and Cox+RSF) based on the C-index. The TCIA NSCLC Radiogenomics cohort was used as the training set, whilst the TCGA LUAD+LUSC cohort and the in-house cohort (Nanyang Central Hospital) served as independent validation sets. **(C)** Radiomic features retained by the Cox+RSF strategy and their corresponding model coefficients. CT-RadScore for each patient was calculated using the selected features weighted by their coefficients. **(D)** Kaplan–Meier survival curves for risk stratification by CT-RadScore (high vs. low). **(E)** Time-dependent ROC curves evaluating the prognostic discrimination of CT-RadScore (1-, 2-, and 3-year AUCs). **(F)** Univariate Cox regression assessing the prognostic value of CT-RadScore. **(G)** Multivariable Cox regression evaluating the independent prognostic value of CT-RadScore after accounting for multicollinearity and adjusting for clinicopathological covariates.

Based on the NSCLC radiogenomics dataset, we first extracted 1,316 quantitative radiomics features using the Pyradiomics in Python. These features were then correlated with the eigengene of the green immune-related WGCNA module, yielding 62 radiomics features that were significantly associated with the immune module (**Figure 2A**; **Supplementary Figure 1F**). These 62 immune-related features were subsequently incorporated into a machine learning–based integrative modelling framework to derive an immune-related CT radiomics signature. We compared three modelling strategies: an elastic-net–penalised Cox model (COX+ENet), a LASSO-penalised Cox model (COX+LASSO) and a Cox model combined with random survival forest (COX+RSF). As shown in **Figure 2B**, the COX+RSF approach consistently achieved the greatest discriminative performance across all cohorts. In the TCIA-NSCLC cohort, the COX+RSF model attained a C-index of 0.791, outperforming COX+ENet (C-index = 0.662) and COX+LASSO (C-index = 0.649). A similar pattern was observed in the TCGA-LUAD+LUSC cohort, where COX+RSF achieved a C-index of 0.729 compared with 0.553 and 0.564 for COX+ENet and COX+LASSO, respectively. In the in-house NSCLC cohort from Nanyang Central Hospital, COX+RSF again provided the best prognostic accuracy, with a C-index of 0.844 versus 0.801 and 0.792 for COX+ENet and COX+LASSO. Collectively, these results demonstrate that the COX+RSF-based model offers superior and robust prognostic performance, and it was therefore adopted as the final immune-related CT radiomics signature for downstream analyses.

### 2.5 Evaluation of the constructed immune-related CT radiomics signature

The final COX+RSF model identified an immune-related CT radiomics signature (CT-RadScore) consisting of 12 non-redundant features with non-zero coefficients (**Figure 2C**). These features encompassed a spectrum of texture, shape, and first-order metrics derived from multi-scale, multi-filtered CT imaging. Positive coefficients were attributed to features such as log-sigma-5-0-mm-3D_glszm_ZoneEntropy, log-sigma-4-0-mm-3D_glszm_ZoneEntropy, and log-sigma-5-0-mm-3D_glszm_LowGrayLevelZoneEmphasis, which capture tumour heterogeneity and textural complexity. Additional positively weighted features included original_shape_Maximum2DDiameterSlice and wavelet-LLL_glszm_GrayLevelVariance, both of which contribute to the assessment of tumour size and internal grey-level variation. Conversely, negative coefficients were associated with features such as wavelet-LLL_glcm_ClusterProminence and log-sigma-4-0-mm-3D_glrlm_RunEntropy, alongside first-order descriptors like log-sigma-5-0-mm-3D_firstorder_Range and log-sigma-4-0-mm-3D_firstorder_Range, reflecting tumour density and structural uniformity. Collectively, these findings underscore that CT-RadScore encapsulates a multi-dimensional representation of immune-related tumour heterogeneity, where higher scores correlate with more aggressive disease, whilst lower scores are indicative of a more favourable clinical outcome.

Patients were subsequently stratified into high- and low-risk groups according to the CT-RadScore. In all three cohorts, high-risk patients had markedly worse overall survival than those in the low-risk group (**Figure 2D**). In the TCIA-NSCLC training cohort, the separation between the two groups was pronounced (log-rank P < 0.0001). Consistent results were observed in the TCGA-LUAD+LUSC validation cohort and in the independent in-house NSCLC cohort from Nanyang Central Hospital (both log-rank P < 0.0001 and P = 0.0017, respectively), underscoring the robustness of the signature across different populations and imaging platforms.

Time-dependent receiver operating characteristic analysis further demonstrated the favourable discriminative capacity of the CT-RadScore (**Figure 2E**). In the TCIA-NSCLC cohort, the CT-RadScore achieved 1-, 2- and 3-year AUCs of 0.777, 0.813 and 0.812, respectively. In the TCGA-LUAD+LUSC cohort, the corresponding AUCs were 0.781, 0.733 and 0.768, whilst in the in-house cohort they reached 0.748, 0.840 and 0.825. These findings indicate that the immune-related CT radiomics signature provides stable short- and intermediate-term survival prediction.

To determine whether CT-RadScore offers prognostic information beyond conventional clinicopathological variables, we performed univariate and multivariate Cox regression analyses in each cohort (**Figures 2F, G**). In univariate analyses, CT-RadScore was strongly associated with overall survival, with hazard ratios substantially greater than those of established factors such as AJCC stage, T/N/M stage and histologic grade. Importantly, after adjustment for age, sex, tumour stage and other available clinical covariates, CT-RadScore remained an independent adverse prognostic factor across datasets, whereas several traditional variables lost statistical significance. Collectively, these results demonstrate that the constructed immune-related CT radiomics signature not only reflects underlying immune-related imaging phenotypes but also confers independent and clinically meaningful prognostic value in patients with NSCLC.

### 2.6 Application of CT-RadScore in immune checkpoint inhibitor therapy

**Figure 3:**
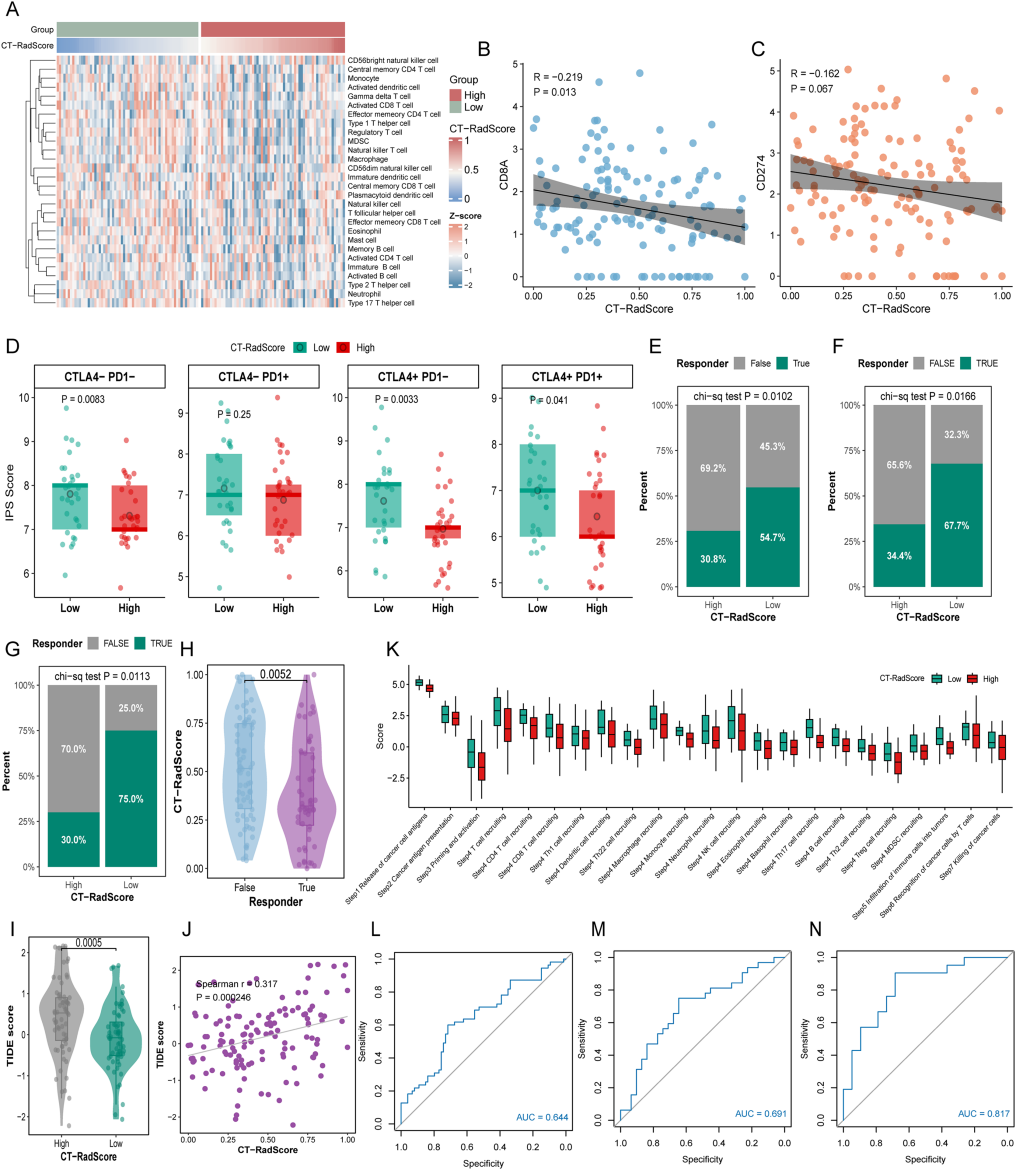
Association of CT-RadScore with the tumour immune contexture and predicted benefit from immune-checkpoint blockade. **(A)** Heat map of inferred immune-cell infiltration profiles in the CT-RadScore low and high groups. **(B, C)** Correlation between CT-RadScore and the expression of CD8A **(B)** and CD274 **(C, D)** Immunophenoscore (IPS) comparisons between CT-RadScore low and high groups under four immunotherapy-relevant settings (CTLA4−/PD1−, CTLA4−/PD1+, CTLA4+/PD1− and CTLA4+/PD1+). **(E–G)** Proportions of predicted immunotherapy responders and non-responders stratified by CT-RadScore group in the TCIA **(E)**, TCGA **(F)** and in-house **(G)** cohorts. **(H)** Distribution of CT-RadScore in responders versus non-responders. **(I, J)** TIDE score comparison between CT-RadScore groups **(I)** and correlation between CT-RadScore and TIDE score **(J, K)** Cancer-immunity cycle activity (TIP scores) across multiple steps, compared between CT-RadScore low and high groups. **(L–N)** Receiver operating characteristic (ROC) curves evaluating CT-RadScore for predicting immunotherapy response in the TCIA **(L)**, TCGA **(M)** and in-house **(N)** cohorts.

Given that the CT-RadScore was developed based on immune-related radiomic features, we hypothesised that variations in CT-RadScore might correlate with differences in immune characteristics and therapeutic response. Immune cell infiltration analysis revealed a significant inverse correlation between CT-RadScore and the abundance of immune cell infiltration (**Figure 3A**). Further correlation analysis demonstrated that higher expression levels of CD8A and CD274 were associated with lower CT-RadScore values (**Figure 3B**), suggesting that CT-RadScore may reflect distinct immune microenvironment features that modulate the response to immunotherapy. The results in **Figure 3D** illustrate the relationship between CT-RadScore and the immune checkpoint inhibitor response, as measured by the IPS score, across different immune checkpoint combinations. In the CTLA4−PD1− group, a significant difference in IPS scores was observed between the low and high CT-RadScore groups (P = 0.0083), with higher IPS scores in the low CT-RadScore group. Conversely, in the CTLA4−PD1+ group, no significant difference was found between the low and high CT-RadScore groups (P = 0.25). However, in the CTLA4+PD1− group, the low CT-RadScore group exhibited significantly higher IPS scores compared to the high CT-RadScore group (P = 0.0033). Similarly, in the CTLA4+PD1+ group, a significant difference was observed (P = 0.041), with lower IPS scores in the high CT-RadScore group.

The results shown in **Figures 3E–G** highlight the significant association between CT-RadScore and immune response to immunotherapy. In the NSCLC radiogenomics dataset (**Figure 3E**), derived from TIDE analysis, patients with low CT-RadScore demonstrated a higher proportion of responders (54.7%) compared to those with high CT-RadScore (30.8%), with a significant difference (P = 0.0102). Similarly, in the TCGA-LUAD+LUSC cohort (**Figure 3F**), also evaluated by TIDE, 67.7% of patients with low CT-RadScore were responders, whereas only 34.4% of those with high CT-RadScore responded to treatment, showing a significant correlation (P = 0.0166). In contrast, real-world data from Nanyang Central Hospital (**Figure 3G**) revealed that 75% of patients with low CT-RadScore were responders, compared to 30% in the high CT-RadScore group, with a significant difference (P = 0.0113). These findings consistently demonstrate that lower CT-RadScore values are associated with improved immunotherapy response across different NSCLC datasets. The CT-RadScore of immune responders was significantly lower than that of non-responders (**Figure 3H**; **Supplementary Figures 2B, D**). The TIDE scores of the low CT-RadScore group were significantly lower than those of the high CT-RadScore group (**Figure 3I**; **Supplementary Figure 2A**). Furthermore, a significant positive correlation between CT-RadScore and TIDE score was observed (**Figure 3J**; **Supplementary Figure 2C**).

Profiling the anti-cancer immune response across the seven-step Cancer-Immunity Cycle reveals clear distinctions between the low and high CT-RadScore groups (**Figure 3K**). The low CT-RadScore group exhibited significantly higher scores at multiple stages, particularly in immune cell trafficking (Step 4), encompassing CD4+ T cells, CD8+ T cells, Th1 and Th2 cells, dendritic cells, macrophages, and NK cells. This group also showed enhanced immune cell infiltration (Step 5) and improved T cell recognition of cancer cells (Step 6). In contrast, the high CT-RadScore group displayed lower scores across most steps, including antigen presentation (Step 2), immune cell recruitment (Step 4), and cancer cell killing by immune cells (Step 7). These results indicate a diminished antitumour immune response in the high CT-RadScore group, characterised by impaired immune cell activation, recruitment, and cancer cell recognition.

The ROC curves for CT-RadScore in diagnosing immunotherapy response versus non-response are presented across three datasets. In the NSCLC radiogenomics dataset (**Figure 3L**), the AUC of 0.644 demonstrates a good level of discriminatory power, indicating that CT-RadScore can reliably distinguish between responders and non-responders. In the TCGA-LUAD+LUSC cohort (**Figure 3M**), the AUC increased to 0.691, reflecting an even stronger ability to predict treatment response. Finally, in the real-world data from Nanyang Central Hospital (**Figure 3N**), the AUC reached 0.817, showcasing an excellent predictive capacity for identifying immunotherapy responders.

### 2.7 The utility of CT-RadScore in drug response

We assessed the value of the CT-RadScore in predicting therapeutic response to various drugs. Significant differences in drug efficacy, as indicated by IC_50_ values, were observed across the CT-RadScore groups, with lower IC_50_ values (indicating increased drug sensitivity) in the high CT-RadScore group for Paclitaxel, Gefitinib, and Carboplatin, all first-line treatments for NSCLC. Notably, Gefitinib (r = -0.31, p = 0.0134) and Paclitaxel (r = -0.27, p = 0.0305) demonstrated significant negative correlations with CT-RadScore, supporting its potential as a predictor of response to these frontline therapies. Furthermore, the CT-RadScore was also associated with response to emerging targeted therapies, such as Zorifertinib (r = -0.27, p = 0.0358), although the correlations were less robust. While Crizotinib and Bemenetnib showed weaker and non-significant correlations, these findings collectively suggest that the CT-RadScore could serve as a valuable tool for predicting drug sensitivity across a spectrum of therapies, thereby enhancing personalised treatment strategies in NSCLC (**Supplementary Figures 3A, B**).

### 2.8 Functional pathway differential analysis under CT-RadScore stratification

**Figure 4:**
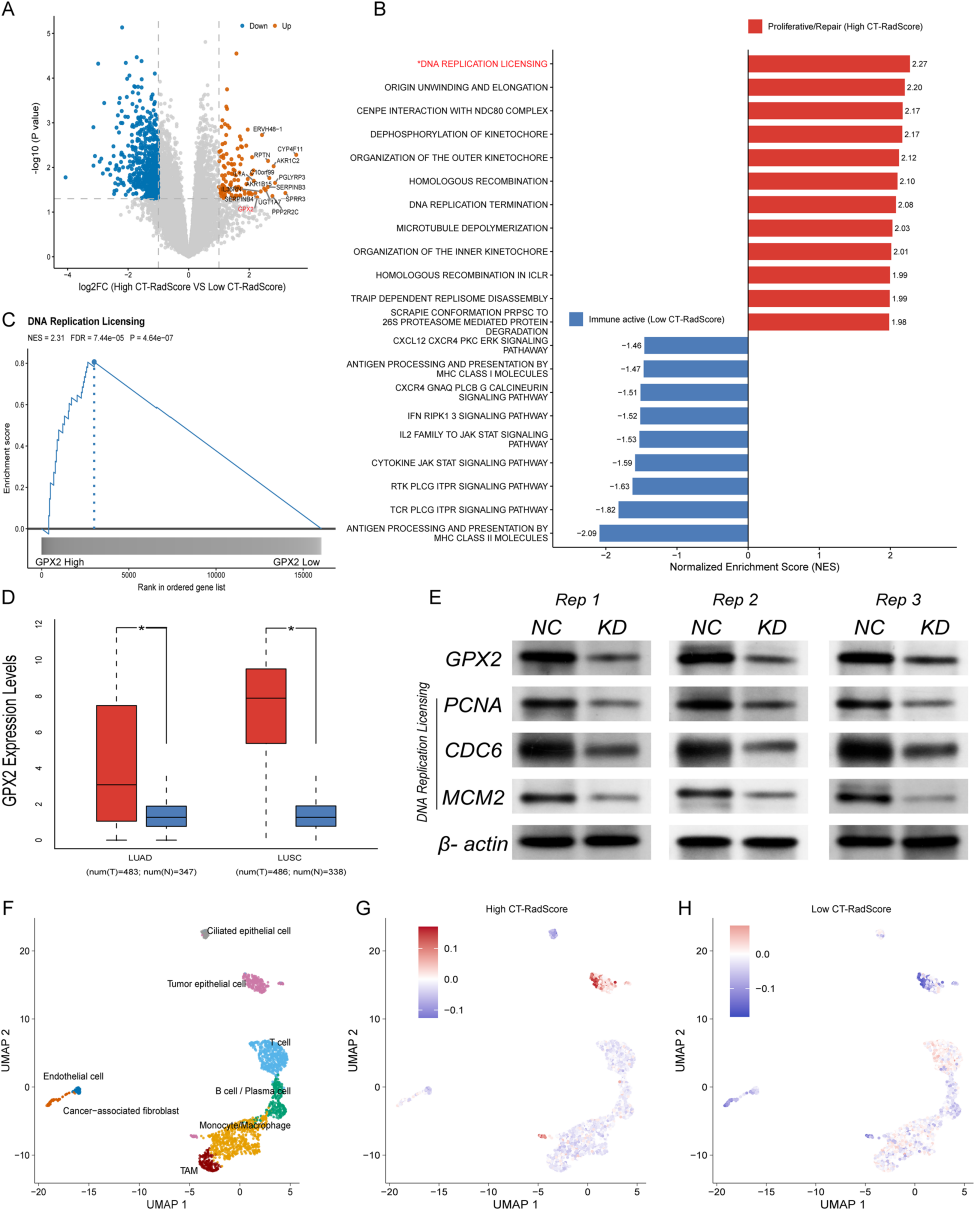
Mechanistic dissection of biological differences between high and low CT-RadScore tumours. **(A)** Volcano plot of differentially expressed genes between the high and low CT-RadScore groups. **(B)** Gene set enrichment analysis (GSEA) comparing pathway activity between CT-RadScore groups. Pathways enriched in the high CT-RadScore group (proliferative/repair programme) and those enriched in the low CT-RadScore group (immune-active programme) are ranked by normalised enrichment score (NES). **(C)** GSEA enrichment plot for the DNA replication licensing pathway, indicating upregulation of this programme in the GPX2-high group. **(D)** GPX2 expression in TCGA lung adenocarcinoma (LUAD) and lung squamous cell carcinoma (LUSC), comparing tumour tissues with adjacent normal tissues. **(E)** Immunoblot validation of GPX2 and replication licensing–related proteins (PCNA, CDC6 and MCM2) following GPX2 knockdown (KD) compared with negative control (NC); β-actin serves as a loading control. **(F)** UMAP projection of single-cell transcriptomes annotated by major cell types. **(G, H)** Feature plots showing the expression levels of two CT-RadScore signature genes in tumours with high **(G)** versus low **(H)** CT-RadScore, highlighting distinct expression patterns across cellular populations.

The volcano plot (**Figure 4A**) illustrates the differential gene expression between high CT-RadScore and low CT-RadScore groups. A clear separation is observed with upregulated genes (marked in orange) and downregulated genes (marked in blue).

In-depth pathway enrichment analysis (**Figure 4B**) highlighted distinct molecular pathways enriched in the high and low CT-RadScore groups. In the high CT-RadScore group, proliferative and repair-associated pathways were prominently enriched, with the most notable being DNA replication licensing (NES = 2.27), followed by origin unwinding and elongation (NES = 2.20) and homologous recombination (NES = 2.12). These findings indicate a heightened state of cellular proliferation and repair in patients with higher CT-RadScore, potentially reflecting an enhanced ability to respond to DNA damage and proliferate under stress conditions. In contrast, the low CT-RadScore group was characterised by the enrichment of immune-related pathways, such as antigen processing and presentation by MHC class I molecules (NES = -2.09) and CXCR4-GNAQ-PLCB-G-calcineurin signalling pathway (NES = -1.82). These results suggest an active immune response in the low CT-RadScore group, with an emphasis on antigen presentation and immune cell signalling pathways, pointing to a more robust immune activation in patients with lower CT-RadScore.

### 2.9 Elevated GPX2 expression in high CT-RadScore is associated with activation of the DNA replication licensing pathway

GSEA revealed that elevated GPX2 expression was strongly associated with activation of the DNA replication licensing pathway in NSCLC. As shown in **Figure 4C**, tumours with high GPX2 expression exhibited markedly stronger enrichment of this pathway (NES = 2.31, FDR = 7.44e-05, P = 4.64e-07), suggesting a close link between GPX2 upregulation and proliferative programs governing DNA replication control. Consistent with this finding, transcriptomic profiling based on RNA-seq demonstrated that GPX2 expression was significantly increased in NSCLC tissues compared with normal lung tissues (**Figure 4D**). Notably, correlation analysis further revealed that GPX2 expression was positively associated with CT-RadScore, and this relationship reached statistical significance, as illustrated in the scatter plot (**Supplementary Figures 4A, B**). Together, these findings suggest that GPX2 upregulation in high CT-RadScore may be linked to radiomics-defined tumour phenotypes characterised by enhanced DNA replication activity.

### 2.10 Knockdown of GPX2 impairs DNA replication licensing

Western blot was performed to validate the effects of GPX2 knockdown on key DNA replication markers in A549 cells. As demonstrated by our experimental data, shRNA-mediated knockdown of GPX2 (KD) led to a marked decrease in CDC6, MCM2 and PCNA protein expression relative to the negative control (NC) (**Figure 4E**).

### 2.11 Single-cell profiling reveals divergent gene expression in high and low CT-RadScore groups

Single-cell RNA sequencing analysis uncovered marked transcriptional divergence between high and low CT-RadScore groups. The tumour microenvironment was resolved into distinct cellular populations, including tumour epithelial cells, ciliated epithelial cells, T cells, B/plasma cells, monocytes/macrophages, tumour-associated macrophages (TAMs), cancer-associated fibroblasts, and endothelial cells (**Figure 4F**; **Supplementary Figure 5A**). Mapping CT-RadScore-associated marker genes onto this cellular landscape revealed strikingly different expression patterns between groups. In the high CT-RadScore cohort, the top characteristic genes were predominantly enriched in tumour epithelial cells, exhibiting strong expression signals within this compartment (**Figure 4G**), suggesting that CT-RadScore–related transcriptional programs are primarily driven by malignant epithelial populations. Conversely, marker genes characterising the low CT-RadScore group were largely enriched within immune cell lineages, including T cells, B cells, and monocytes/macrophages (**Figure 4H**), indicative of a more immune-active microenvironment. These findings highlight a fundamental biological contrast between the two groups: high CT-RadScore tumours are characterised by tumour epithelial–dominant gene expression programs, whereas low CT-RadScore tumours exhibit prominent immune cell–associated transcriptional signatures.

## 3 Discussion

NSCLC remains a leading cause of cancer-related mortality worldwide, and phenotypes of the tumour immune microenvironment that confer poor prognosis and pronounced heterogeneity in therapeutic responses continue to represent a major barrier to improving clinical outcomes in NSCLC (19, 20). In this study, we developed and validated an immune-related CT-based radiomics signature (CT-RadScore) that links quantitative imaging features to patterns of immune cell infiltration. Across multiple independent cohorts of patients with NSCLC, CT-RadScore enabled robust risk stratification and identified patient subgroups deriving differential benefit from systemic therapies, including immune checkpoint inhibitors, chemotherapy, and targeted therapies. As an imaging biomarker capturing the immune landscape of the TME, CT-RadScore provides both prognostic information and treatment-specific predictive value, and may facilitate refined risk stratification and the personalised application of immunotherapy, chemotherapy and targeted therapy in NSCLC.

Our consensus clustering analysis identified two distinct immune subtypes (immune-cold C1 and immune-hot C2) using ssGSEA-derived immune cell infiltration data, with robustness confirmed across seven independent immune analysis algorithms (TIMER, QUANTISEQ, MCPCOUNTER, xCell, EPIC, CIBERSORT, ESTIMATE). This bimodal immune stratification aligns with established TME classification frameworks but extends prior work by linking immune phenotypes to CT imaging features (21). The green module—strongly correlated with immune clusters (GS–MM correlation = 0.91)—was enriched for pathways central to innate and adaptive immunity, including cytokine–cytokine receptor interaction, chemokine signalling, antigen processing and presentation, and NF-κB/TNF cascades. These findings underscore that the hub genes of the green module orchestrate immune-cell recruitment, activation and effector function within the TME, and provide functional support for the biological rationale of using this immune-related module as a molecular anchor to select correlated radiomic features for subsequent model development. Notably, functional pathway analysis stratified by CT-RadScore revealed divergent biological programs: high CT-RadScore tumours were characterised by proliferative/DNA repair pathways (DNA replication licensing, homologous recombination), whilst low CT-RadScore tumours exhibited enrichment of immune-active pathways (MHC class I antigen presentation, CXCR4-mediated immune signalling). Single-cell RNA sequencing further clarified this dichotomy: high CT-RadScore tumours were dominated by tumour epithelial cell transcriptional signatures, whereas low CT-RadScore tumours displayed prominent immune cell (T/B cells, monocytes/macrophages) gene expression—a finding that aligns with the inverse correlation between CT-RadScore and immune cell infiltration (e.g., CD8A+ T cells) and highlights that CT-RadScore inherently captures the balance between tumour cell proliferation and antitumour immunity, two key determinants of clinical outcomes.

We further identified GPX2 as a potential mechanistic mediator of the CT-RadScore: in high CT-RadScore tumours, GPX2 expression was significantly upregulated and positively associated with activation of the DNA replication licensing pathway. Consistent with this association, shRNA-mediated GPX2 knockdown in A549 cells reduced the expression of key replication licensing markers (CDC6, MCM2 and PCNA). GPX2 is a selenium-dependent glutathione peroxidase that detoxifies peroxides and has been implicated in lung adenocarcinoma progression, cisplatin resistance and unfavourable prognosis, as well as in metabolic remodelling of the tumour immune microenvironment and attenuated responses to ICB (22–24). Taken together, these observations support a model in which GPX2-driven redox and proliferation programmes contribute to an immune-quiescent tumour microenvironment, thereby providing a biological rationale for the adverse clinical outcomes associated with high CT-RadScore.

The COX+RSF-derived CT-RadScore (12 non-redundant radiomic features) demonstrated robust prognostic performance across three independent cohorts (TCIA-NSCLC: C-index=0.791; TCGA-LUAD+LUSC: C-index=0.729; in-house: C-index=0.844), with high-risk patients exhibiting significantly worse overall survival (all log-rank P<0.0017). Critically, CT-RadScore remained an independent prognostic factor after adjusting for conventional clinicopathological variables (AJCC stage, T/N/M stage, histologic grade), outperforming established clinical predictors—a distinction that is particularly impactful given the limitations of current prognostic tools in NSCLC, many of which rely on invasive tissue sampling or lack reproducibility across populations (25, 26). Beyond prognosis, CT-RadScore emerged as a powerful predictor of ICI response: low CT-RadScore was consistently associated with higher response rates across datasets (NSCLC radiogenomics: 54.7% vs. 30.8%; TCGA: 67.7% vs. 34.4%; in-house: 75% vs. 30%; all P<0.0166) and lower TIDE scores (a validated surrogate for ICI resistance), with strong discriminatory capacity (in-house AUC = 0.817). Mechanistically, this aligns with impaired antitumour immune cycling in high CT-RadScore tumours—characterised by deficits in antigen presentation, immune cell recruitment, infiltration, and cancer cell killing—and the observed correlation between CT-RadScore and CD8A/CD274 expression further supports its ability to capture immune-active TME features predictive of ICI sensitivity. This interpretation is also supported by the specific immune pathways enriched in low CT-RadScore tumours. In particular, enrichment of MHC class I antigen presentation-related programmes is biologically relevant to immune checkpoint blockade, because effective presentation of tumour antigens to cytotoxic T cells is a prerequisite for T-cell priming and recognition of malignant cells; conversely, defects in antigen-processing and presentation machinery have been associated with resistance to PD-1/PD-L1 blockade in NSCLC and other cancers (27). In addition, enrichment of cytokine–cytokine receptor interaction and chemokine-related signalling suggests a tumour microenvironment more permissive to immune-cell recruitment, trafficking and intercellular communication, all of which are critical for establishing a T cell-inflamed phenotype that is more likely to respond to ICIs (28). The observed enrichment of CXCR4-mediated immune signalling may also be relevant in this context. Although CXCR4 signalling can have context-dependent pro-tumour or immunosuppressive effects, it is also involved in leukocyte trafficking, immune synapse formation and T-cell functional organisation (29); therefore, in our dataset, its enrichment in low CT-RadScore tumours may reflect a more immunologically engaged microenvironment rather than a purely tumour-promoting programme. Likewise, NF-κB/TNF-related pathways may contribute to immunotherapy responsiveness in a context-dependent manner by regulating inflammatory cytokine production, immune-cell activation and PD-L1 expression, thereby shaping both immune priming and adaptive immune resistance (30). Taken together, these pathway-level findings strengthen the biological plausibility that low CT-RadScore tumours represent a more immune-permissive and therapeutically responsive state, whereas high CT-RadScore tumours are characterised by impaired antigen presentation and reduced immune engagement, which may limit the efficacy of ICIs. These results address an unmet need in immunotherapy: identifying responders without reliance on tissue-based PD-L1 testing, which is limited by sampling bias and accessibility. CT-RadScore also demonstrated utility in predicting drug response, with high-risk patients exhibiting increased sensitivity to first-line therapies (Paclitaxel, Gefitinib and Carboplatin) as indicated by lower IC_50_ values; the strong negative correlation between CT-RadScore and gefitinib sensitivity (r = −0.31, P = 0.0134) is particularly clinically relevant, as EGFR-mutant NSCLC often requires biomarkers to guide TKI selection. Collectively, these data suggest that CT-RadScore may serve as a theranostic tool—providing both prognostic stratification and support for personalised treatment selection (for example, prioritising ICIs in low-risk and targeted therapies or chemotherapy in high-risk patients)—and has the potential to refine current strategies for NSCLC management. From a translational perspective, several potential clinical application scenarios of CT-RadScore merit consideration. First, in patients with advanced NSCLC being evaluated for first-line systemic treatment, CT-RadScore may serve as a non-invasive tool to identify those more likely to benefit from immune checkpoint blockade, particularly when tumour tissue is limited, PD-L1 testing is unavailable, or biopsy samples may not adequately capture spatial heterogeneity of the tumour immune microenvironment. In this setting, a low CT-RadScore could support prioritisation of immunotherapy-based strategies, whereas a high CT-RadScore may suggest a less immune-responsive state and prompt consideration of alternative approaches, such as chemotherapy or molecularly targeted therapy when clinically indicated. Second, in patients with potentially targetable oncogenic alterations, CT-RadScore may provide complementary information beyond genomic testing by helping to distinguish tumours with predominantly proliferative versus immune-active phenotypes, thereby informing therapeutic prioritisation or sequencing. Third, CT-RadScore could potentially be incorporated into real-world multidisciplinary decision-making workflows as an imaging-derived adjunct to established clinicopathological and molecular biomarkers. Because it is based on routinely acquired CT imaging, CT-RadScore could be calculated before treatment initiation and integrated with clinical stage, histology, PD-L1 expression, and genomic alterations to support risk-adapted treatment selection. Although prospective validation is still required, such a workflow may be particularly valuable in settings where repeated tissue acquisition is impractical, and may ultimately facilitate more precise and accessible treatment stratification in NSCLC.

While several radiomic signatures have been proposed for NSCLC prognosis and ICI prediction, our study offers three distinct advantages. First, its immune-centric design sets it apart from conventional radiomic models that focus on tumour morphology alone: CT-RadScore is directly anchored to immune infiltration patterns, ensuring biological relevance to immunotherapy response. Second, its robust performance across TCIA, TCGA, and an independent in-house cohort (with different imaging platforms) confirms generalizability—a critical prerequisite for clinical translation. Third, its dual predictive utility (simultaneous prediction of ICI and drug response) addresses the need for integrated biomarkers in an era of personalised therapy. Importantly, prior immune radiomic studies have often lacked mechanistic validation, whereas our integration of WGCNA, single-cell RNA-seq, and functional experiments (GPX2 knockdown) provides a molecular framework linking radiomic features to TME biology; for example, CT-RadScore’s texture/shape features (e.g., log-sigma-5-0-mm-3D_glszm_ZoneEntropy, original_shape_Maximum2DDiameterSlice) correlate with immune cell infiltration and tumour proliferation, explaining their prognostic/predictive power and distinguishing our work from descriptive radiomic studies to strengthen its clinical relevance.​ In addition, the association between radiomic heterogeneity and the tumour immune microenvironment may provide an important biological basis for interpreting CT-RadScore. Texture-based radiomic features quantify spatial variations in voxel intensity and structural complexity within the tumour, and are therefore considered imaging surrogates of intratumoural heterogeneity. Because the immune microenvironment is itself spatially heterogeneous, with regional differences in immune-cell infiltration, stromal composition, necrosis, vascularity and tumour-cell density, radiomic heterogeneity may capture macroscopic consequences of these microscopic immune and stromal patterns (31, 32). Previous studies support this concept: immune-pathology-informed radiomics in NSCLC has shown that a high-heterogeneity/low-intensity CT phenotype is associated with low PD-L1 expression and high CD3+ T-cell infiltration, consistent with an immune-activated state (33); similarly, radiomic signatures have been reported to discriminate immune-inflamed from immune-desert tumours and to correlate with CD8-related immune phenotypes and immunotherapy outcomes (34). These findings suggest that radiomic heterogeneity is not merely a morphological descriptor, but may reflect the spatial organisation and functional state of the tumour immune microenvironment. In this context, the inverse relationship observed in our study between CT-RadScore and immune infiltration further supports the biological plausibility that CT-derived heterogeneity features can serve as non-invasive surrogates of immune contexture in NSCLC.

Despite these strengths, our study has limitations that warrant consideration. First, its retrospective design may introduce selection bias, particularly in the in-house cohort, and prospective validation in a multicentre trial is needed to confirm CT-RadScore’s clinical utility. Second, whilst we evaluated ICI response using TIDE scores and real-world data, the lack of prospective ICI-treated cohorts with paired CT imaging and outcome data limits definitive conclusions about its predictive performance for ICI efficacy. Third, the molecular mechanisms underlying the correlation between radiomic features and immune/TME phenotypes require further investigation—for example, *in vitro* studies to determine how specific imaging features (e.g., textural complexity) correlate with immune cell recruitment or GPX2 expression. Fourth, CT-RadScore was developed using Pyradiomics-derived features, and standardisation of imaging acquisition and feature extraction across institutions is essential for broad clinical adoption. Future research should therefore focus on multiple fronts: prospective validation of CT-RadScore in ICI-treated NSCLC patients to establish its role as a companion diagnostic; integration of CT-RadScore with other biomarkers (e.g., circulating tumour DNA, PD-L1 expression) to improve predictive accuracy; exploring the utility of CT-RadScore in guiding combination therapies (e.g., ICI + targeted therapy) for intermediate-risk patients; extending the signature to other cancer types with distinct immune phenotypes; and developing deep learning models to automate CT-RadScore calculation and enhance clinical feasibility.

In summary, we developed a novel CT-based immune radiomic signature (CT-RadScore) that non-invasively captures TME immune phenotypes and tumour proliferative programs, providing independent prognostic value and robust prediction of ICI and drug response in NSCLC. CT-RadScore’s integration of routine imaging with immune biology addresses critical unmet needs in precision oncology—eliminating reliance on invasive tissue sampling, refining risk stratification, and guiding personalised treatment selection. With prospective validation and standardisation, CT-RadScore has the potential to transform clinical decision-making for NSCLC patients, improving outcomes and reducing the burden of ineffective therapies. The mechanistic insights linking CT-RadScore to GPX2-driven DNA replication and immune cycling also open new avenues for therapeutic targeting, particularly in high-risk patients with immune-cold tumours.

## 4 Materials and methods

### 4.1 Dataset collection and processing

Data were obtained from multiple sources to ensure the comprehensiveness and robustness of the analysis. From the TCIA database, the NSCLC Radiogenomics cohort was retrieved, encompassing CT imaging, gene expression profiles, and clinical information. To maintain data integrity and comparability, cases containing only imaging or gene expression data were excluded, resulting in a final cohort of 129 patients. In addition, 63 patients were included from the TCGA LUAD and LUSC cohorts, for whom CT imaging, gene expression, and clinical data were all available. Moreover, CT images of 40 patients with NSCLC were retrospectively collected from the Department of CT Imaging Diagnosis, Nanyang Central Hospital, using the Radiology Information System. The inclusion criteria were as follows: adequate image quality, histopathological confirmation of NSCLC, availability of TNM staging data, follow-up information, and immunotherapy records.

To minimise batch effects between the LUAD and LUSC cohorts, the “sva” package in R was applied prior to downstream analyses.

### 4.2 Consensus clustering

According to the immune cell infiltration patterns, an unsupervised consensus clustering algorithm with resampling was applied to the NSCLC Radiogenomics cohort using the “ConsensusClusterPlus” package. The optimal number of clusters was selected by jointly considering the consensus matrix, CDF curve, PAC score, and NbClust indices.

### 4.3 Immune cell infiltration

Multiple computational algorithms, including XCELL, TIMER, QUANTISEQ, MCPCOUNTER, ESTIMATE, EPIC, CIBERSORT, and single-sample gene set enrichment analysis (ssGSEA), were employed to comprehensively evaluate the immune cell infiltration landscape in patients with NSCLC.

### 4.4 Weighted gene co-expression network analysis

Co-expression gene networks for the NSCLC Radiogenomics cohort were constructed using the “WGCNA” package in R. An optimal soft-thresholding power (β) was selected to satisfy the scale-free topology criterion. The resulting weighted adjacency matrix was then converted into a topological overlap matrix (TOM), from which the corresponding dissimilarity measure (1 - TOM) was derived. Gene modules were identified using a dynamic tree-cutting algorithm. To determine the modules most strongly associated with immune clusters, the module exhibiting the highest correlation was selected for further analysis. Genes with high gene significance (GS) and module membership (MM) values were defined as immune-related genes.

### 4.5 CT radiomics feature extraction and processing

The CT radiomics processing workflow comprised three principal stages: image preprocessing, tumour delineation, and feature extraction. All CT images were imported in DICOM format into 3D Slicer, where experienced radiologists manually delineated the tumour regions of interest (ROI). Following ROI definition, voxel resampling was performed to standardise voxel dimensions to 1 × 1 × 1 mm³, thereby minimising the influence of inter-patient variability in scanning parameters. High-throughput quantitative features were subsequently extracted using PyRadiomics, covering first-order statistical features (e.g., mean, standard deviation, skewness), morphological features (e.g., volume, surface area), and texture features (e.g., grey-level co-occurrence matrix [GLCM] and grey-level run-length matrix [GLRLM]). Radiomics data preprocessing was conducted in Python, including the removal of missing values and the application of Z-score normalisation to all numerical variables to eliminate the effects of scale discrepancies amongst features on model performance.

### 4.6 Development of a CT radiomic signature using machine learning approaches

To develop an immune-related CT radiomic signature with both accuracy and robustness, we trained three survival models in parallel in the NSCLC Radiogenomics training cohort: random survival forest (RSF), LASSO-Cox, and Elastic Net-Cox (ENet-Cox). We first performed correlation analyses based on immunologic relevance to preselect radiomic features, then applied univariable Cox regression within the training cohort to retain prognostically informative candidates. Using these candidates, RSF, LASSO-Cox, and ENet-Cox models were fitted with hyperparameters optimised by cross-validation. All models underwent external validation in two independent cohorts (TCGA LUAD+LUSC and an in-house dataset). Harrell’s concordance index (C-index) was computed in the training and validation datasets, and the model with the highest cross-dataset mean C-index was designated the optimal model; its selected features and coefficients constituted the radiomic signature. The patient-level composite score, CT-RadScore, was defined as the continuous risk output of the corresponding model and was used for cross-cohort comparisons and downstream statistical analyses. For survival and subgroup analyses, patients were stratified into high and low CT-RadScore groups using the median CT-RadScore value as the cutoff.

### 4.7 Tumour immune dysfunction and exclusion assessment

We employed the TIDE framework to assess the potential responsiveness of our samples to ICB. For implementation, bulk RNA-seq expression matrices served as input, gene expression was normalised, and the TIDE algorithm was run under default settings to obtain a per-sample TIDE score. In downstream analyses, objective ICB response status and the TIDE score were treated as primary readouts; we evaluated their associations with CT-RadScore and compared these metrics across CT-RadScore strata to investigate the relationship between CT-RadScore and potential clinical benefit from ICB.

### 4.8 Tracking tumour immunophenotype

The TIP framework was applied to characterise anti-cancer immunity across the seven steps of the Cancer–Immunity Cycle and to summarise the composition of tumour-infiltrating immune cells at the sample level. Individuals were stratified into high- and low CT-RadScore groups based on the prespecified cut-off. TIP-derived metrics were subsequently compared between these groups to assess immunophenotypic differences associated with CT-RadScore.

### 4.9 Drug sensitivity analysis

The “oncoPredict” package, along with reference drug response databases, was utilised to estimate drug sensitivity for each sample, resulting in predicted IC_50_ values for individual agents. Individuals were stratified into high and low CT-RadScore groups based on the prespecified cut-off, and differences in IC_50_ values between the groups were compared. Additionally, the association between CT-RadScore and IC_50_ was assessed through correlation analysis to evaluate the overall relationship between the score and drug sensitivity.

### 4.10 Establishment of GPX2-knockdown A549 cell lines using lentiviral shRNA

A549 human NSCLC cells were cultured in DMEM with 10% FBS at 37 °C and 5% CO_2_. Lentiviral vectors carrying a GPX2-targeting shRNA (KD) or negative-control shRNA (NC) were obtained from GenePharma (Shanghai, China) and used to infect A549 cells in the presence of polybrene. Stable GPX2-knockdown and control cell lines were selected with puromycin. The shRNA sequence targeting GPX2 was 5′-GCACAACCACCCGGGACTTCA-3′.

### 4.11 Single-cell RNA sequencing analysis

We analysed the NSCLC single-cell RNA-seq dataset GSE117570 using the Seurat package in R. Cell-level quality control was first performed: cells with ≥300 detected features were retained, and cells with ≥5% mitochondrial transcript content were discarded. To remove outliers, the 95th percentile was used as an upper bound, excluding cells whose detected features or UMI counts exceeded the sample-specific 95th percentile thresholds. Subsequently, data were normalised and scaled, cell-cycle scores were regressed out, and batch effects were corrected using Harmony integration. Following the standard Seurat workflow, highly variable genes were identified, PCA was performed for dimensionality reduction, and a shared nearest-neighbour graph was constructed for cell clustering. Cell clusters were visualised using UMAP. Because CT-RadScore is derived from CT radiomic features and cannot be directly calculated in single-cell datasets lacking imaging information, we implemented a gene-expression–based projection strategy to investigate CT-RadScore-associated transcriptional programs at single-cell resolution. Specifically, differential gene expression analysis was first performed between high- and low-CT-RadScore groups in the bulk transcriptomic cohort, and the top 2000 upregulated genes in each group were defined as CT-RadScore-associated gene signatures. These gene signatures were subsequently projected onto the scRNA-seq dataset, and module scores representing the enrichment of high-CT-RadScore and low-CT-RadScore gene signatures were calculated for each cell using the Seurat function AddModuleScore, enabling the characterisation of CT-RadScore-associated transcriptional programs across distinct cellular populations within the tumour microenvironment.

### 4.12 Statistical analysis

All statistical analyses were conducted in R (version 4.5.1). Between-group differences were evaluated using the Wilcoxon rank-sum test for continuous variables and the χ² test for categorical variables. Associations between variables were assessed with Spearman’s rank correlation. All tests were two-sided, and statistical significance was defined as P < 0.05.

## Data Availability

The datasets analysed in this study comprise publicly accessible repositories and an institutional in-house cohort. The NSCLC Radiogenomics imaging dataset was obtained from The Cancer Imaging Archive (TCIA; https://www.cancerimagingarchive.net), the LUAD and LUSC cohorts from The Cancer Genome Atlas (TCGA; https://portal.gdc.cancer.gov/), and transcriptomic profiles from the Gene Expression Omnibus (GEO; https://www.ncbi.nlm.nih.gov/geo) under accession numbers GSE103584 and GSE117570. The in-house NSCLC cohort from Nanyang Central Hospital is not publicly available owing to patient privacy and institutional regulations; de-identified data are available from the corresponding author upon reasonable request and subject to institutional review board approval.
