# Supplementary material for: CT-based immune radiomic signature for prognosis and prediction of immunotherapy and anticancer drug response in NSCLC
Source: Front Immunol. 2026 Apr 29;17:1767389. doi: 10.3389/fimmu.2026.1767389 (PMC13168087; doi:10.3389/fimmu.2026.1767389)

Supplementary Material

# Supplementary Figures

## Supplementary Fig. 1 | Consensus clustering and WGCNA analyses supporting immune subtyping and immune-associated radiomic feature selection.

A, Proportion of ambiguous clustering (PAC) curve across candidate cluster numbers (k). Lower PAC values indicate more stable clustering; the optimal solution was k = 2.

B, Determination of the optimal number of clusters using NBCLUST. The bar plot summarizes recommendations from 26 independent indices.

C, Heat map of immune-cell infiltration patterns comparing the two consensus clusters, quantified using six independent deconvolution algorithms (TIMER, QUANTISEQ, MCP-COUNTER, XCELL, EPIC and CIBERSORT) to assess robustness of immune-infiltration estimates. Values are Z-score scaled.

D, Selection of the soft-thresholding power for WGCNA. The left panel shows the scale-free topology model fit as a function of power, and the right panel shows the corresponding mean connectivity.

E, Eigengene adjacency heat map depicting correlations among module eigengenes and hierarchical clustering of modules.

F, Radiomic features significantly associated with the immune-related module; bar plot shows the significance of the 62 immune-associated radiomic features.


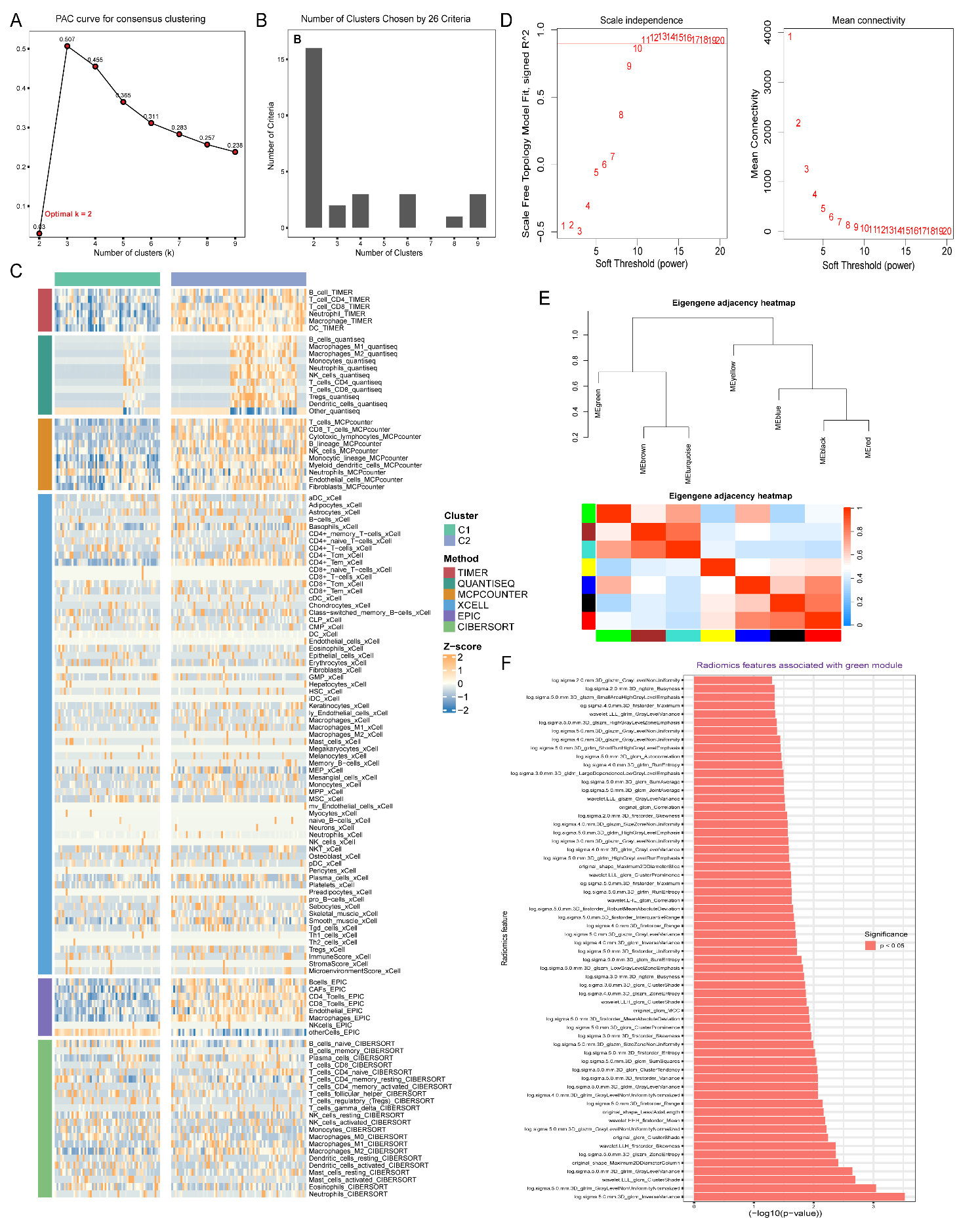


## Supplementary Fig. 2 | CT-RadScore is associated with TIDE and predicted immunotherapy response in external cohorts.

A–C, Analyses in the TCGA cohort. A, Comparison of TIDE scores between the high and low CT-RadScore groups. B, Distribution of CT-RadScore stratified by predicted immunotherapy response status (responder versus non-responder). C, Correlation between CT-RadScore and TIDE score.

D, Analysis in the in-house cohort. Distribution of CT-RadScore stratified by immunotherapy response status (responder versus non-responder).


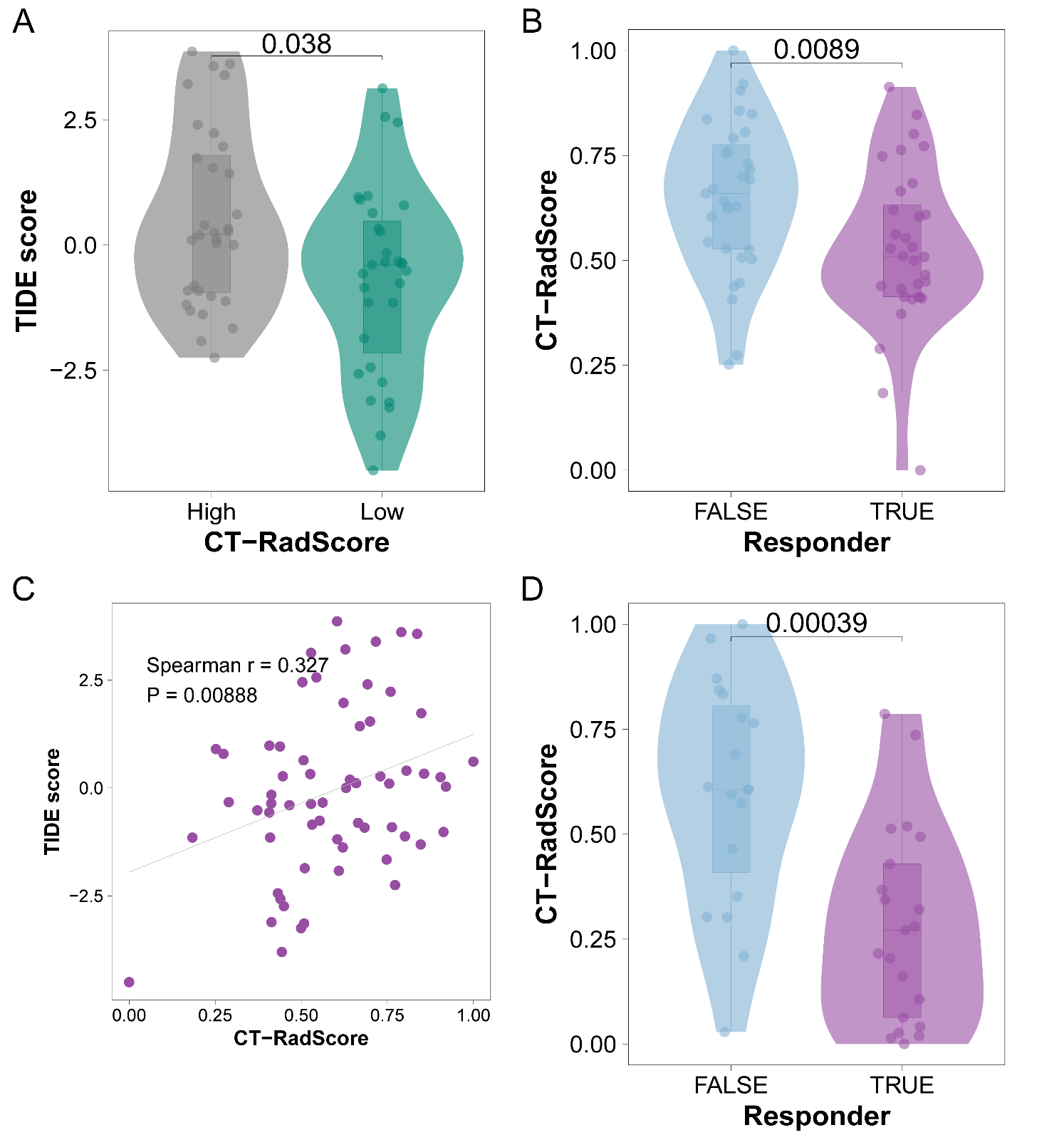


## Supplementary Fig. 3 | CT-RadScore is associated with predicted sensitivity to anticancer agents.

A, Predicted drug sensitivity (as indicated in each panel) compared between the low and high CT-RadScore groups, including paclitaxel, gefitinib, crizotinib, bemcentinib, zorifertinib and carboplatin. Box plots show the median and interquartile range, with individual samples overlaid.

B, Correlation between CT-RadScore and predicted drug sensitivity for the same agents. Lines indicate linear fits and shaded areas denote 95% confidence intervals; correlation coefficients (r) and two-sided P values are shown in each panel.


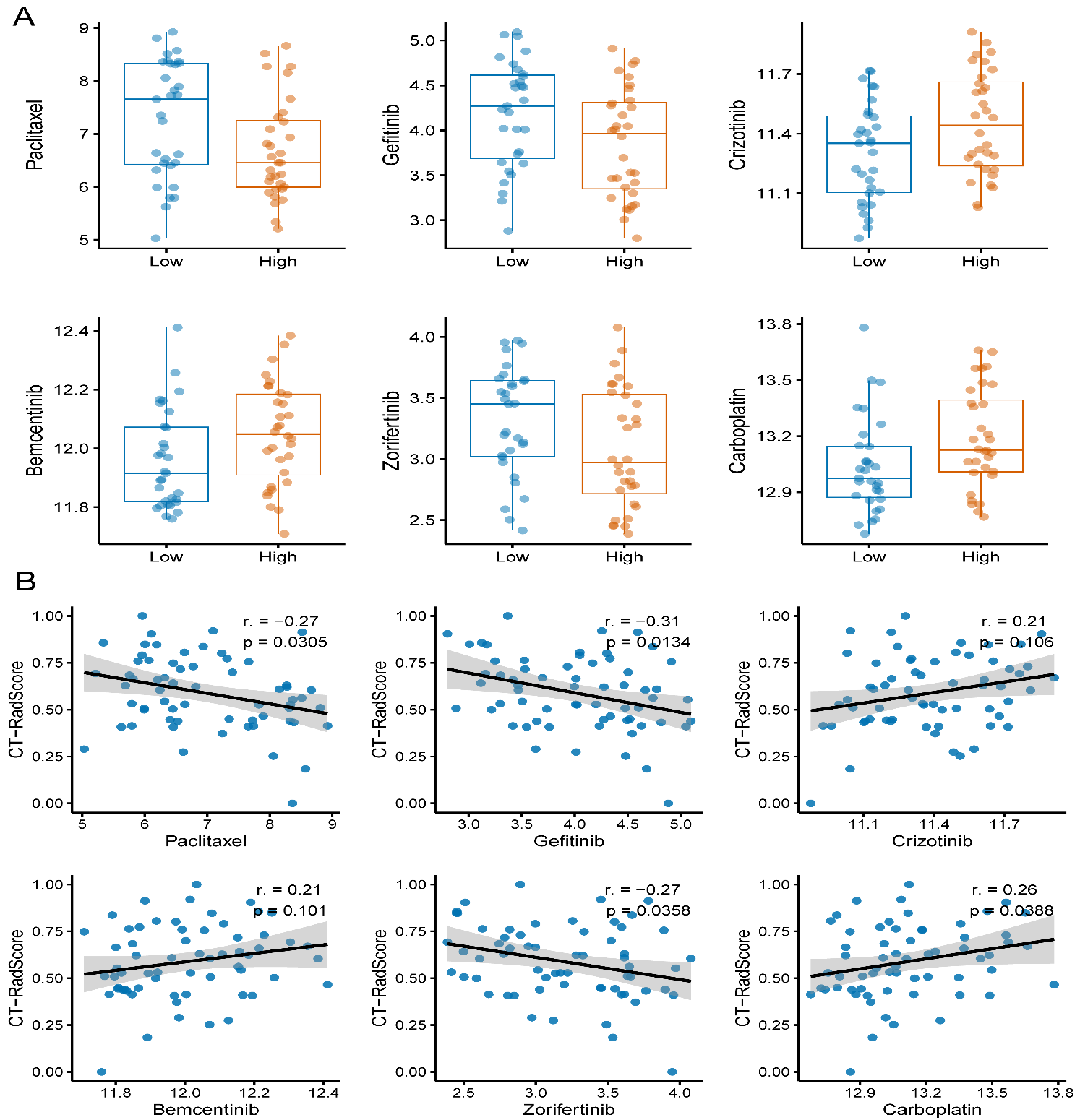


A


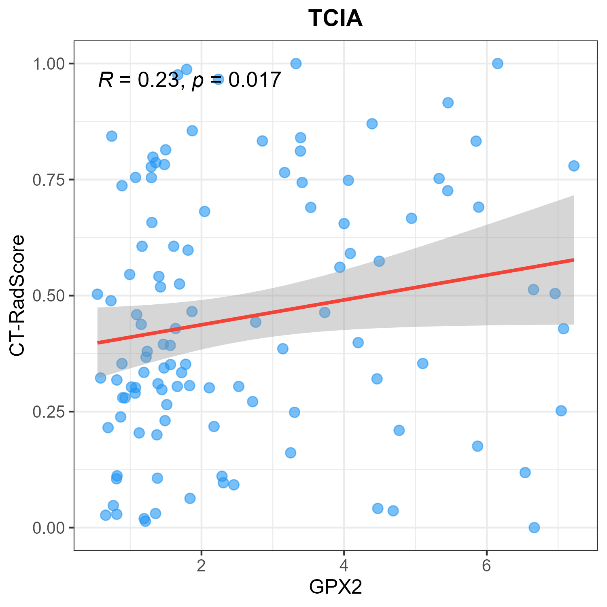


B


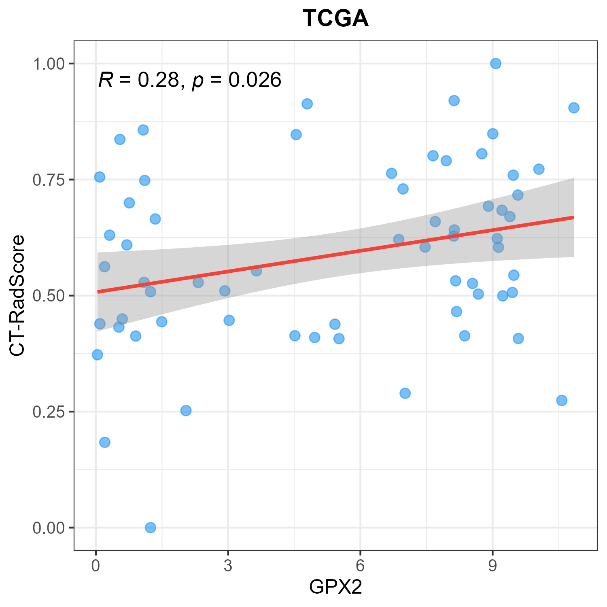


## Supplementary Fig. 4 | Correlation between GPX2 expression and CT-RadScore.

A & B, Scatter plot analysis demonstrated that GPX2 expression was positively correlated with CT-RadScore in both the TCIA and TCGA cohorts.

## Supplementary Fig. 5 | Marker gene expression defining major cell populations in the single-cell RNA-seq dataset.

A, Dot plot showing the expression of canonical marker genes across annotated cell types. Dot colour indicates the average scaled expression level, and dot size indicates the percentage of cells expressing each gene within a given cell type. Markers include B cell/plasma cell genes (CD79A, IGKC, IGHG1), cancer-associated fibroblast genes (TAGLN, SPARC), ciliated epithelial genes (DNAH12, CFAP52), endothelial genes (EFNB2, EDN1), monocyte/macrophage genes (CSF1R, FCN1, IL1B), T cell genes (CD3D, CD3E, CD2), tumour-associated macrophage genes (MARCO, CCL18), and tumour epithelial genes (CEACAM6, SFTPA1, NAPSA).


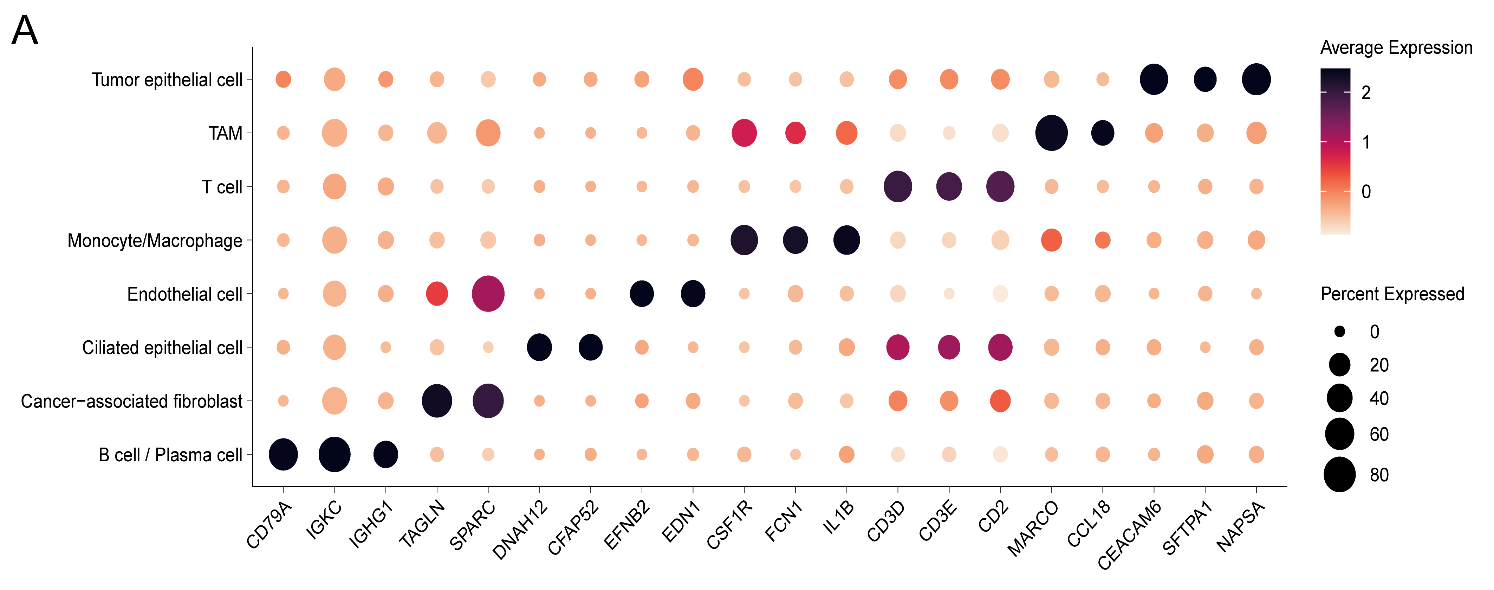


# scRNA-seq analysis qc script

rm(list = ls())

library(tidyverse)

library(data.table)

library(Seurat)

library(harmony)

library(future)

library(ggplot2)

plan('multisession', workers = 8)

options(future.globals.maxSize = 30 * 1024^3)

set.seed(20251026)

file_path <- list.files('raw_data/', full.names = TRUE)

sc_list <- list()

for (fp in file_path) {

dt <- fread(fp)

gene_col <- names(dt)[1]

mat <- dt %>%

as.data.frame() %>%

tibble::column_to_rownames(gene_col)

rn <- rownames(mat)

mat <- as.matrix(mat)

storage.mode(mat) <- 'numeric'

rownames(mat) <- make.unique(rn)

base <- basename(fp)

sample_id <- stringr::str_extract(base, '(?<=_)P\\d+(?=_)')

seu <- CreateSeuratObject(counts = mat, min.cells = 3, min.features = 200)

seu$orig.ident <- sample_id

seu <- RenameCells(seu, add.cell.id = sample_id)

sc_list[[sample_id]] <- seu

}

mt_pattern <- '^MT-'

min_features <- 300

mt_pct_max <- 5

max_features_q <- 0.95

max_counts_q <- 0.95

qc_one <- function(obj) {

obj[['percent.mt']] <- PercentageFeatureSet(obj, pattern = mt_pattern)

feat_upper <- quantile(obj$nFeature_RNA, max_features_q, na.rm = TRUE)

coun_upper <- quantile(obj$nCount_RNA, max_counts_q, na.rm = TRUE)

subset(

obj,

subset = nFeature_RNA > min_features &

nFeature_RNA < feat_upper &

percent.mt < mt_pct_max &

nCount_RNA < coun_upper

)

}

sc_list <- lapply(sc_list, qc_one)

s.genes <- Seurat::cc.genes.updated.2019$s.genes

g2m.genes <- Seurat::cc.genes.updated.2019$g2m.genes

seu <- Reduce(function(a, b) merge(a, b), sc_list)

seu <- JoinLayers(seu, assay = 'RNA', layers = 'counts')

seu[['percent.mt']] <- PercentageFeatureSet(seu, pattern = mt_pattern)

seu <- NormalizeData(seu, normalization.method = 'LogNormalize', scale.factor = 1e4, verbose = FALSE)

seu <- FindVariableFeatures(seu, selection.method = 'vst', nfeatures = 2500, verbose = FALSE)

seu <- CellCycleScoring(seu, s.features = s.genes, g2m.features = g2m.genes, set.ident = FALSE)

seu <- ScaleData(

seu,

features = VariableFeatures(seu),

vars.to.regress = c('S.Score', 'G2M.Score', 'percent.mt'),

verbose = FALSE

)

seu <- RunPCA(seu, features = VariableFeatures(seu), verbose = FALSE)

seu$orig.ident <- factor(seu$orig.ident)

ElbowPlot(seu)

seu <- RunHarmony(

object = seu,

group.by.vars = 'orig.ident',

dims = 1:10,

verbose = FALSE

)

seu <- FindNeighbors(seu, reduction = 'harmony', dims = 1:10, verbose = FALSE)

seu <- FindClusters(seu, resolution = 0.2, verbose = FALSE)

seu <- RunUMAP(seu, reduction = 'harmony', dims = 1:10, verbose = FALSE)

DimPlot(seu, reduction = 'umap')

saveRDS(seu, file = 'seu.rds')

seu = readRDS('seu.rds')

markers = FindAllMarkers(seu, only.pos = TRUE, min.pct = 0.25, logfc.threshold = 0.25)

top_markers = markers %>%

group_by(cluster) %>%

arrange(desc(avg_log2FC)) %>%

slice_head(n = 10) %>%

ungroup()

write_tsv(top_markers, 'cluster_marker.tsv')

# CT imaging characteristics across different CT-RadScore groups


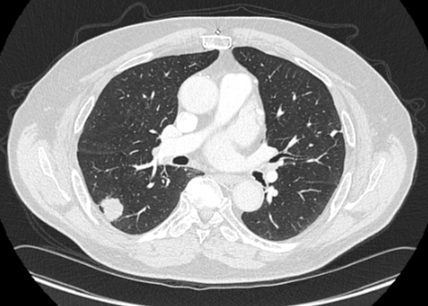


**High CT-RadScore**

**
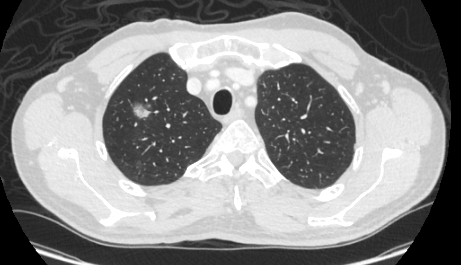
**

**Low CT-RadScore**

# Workflow


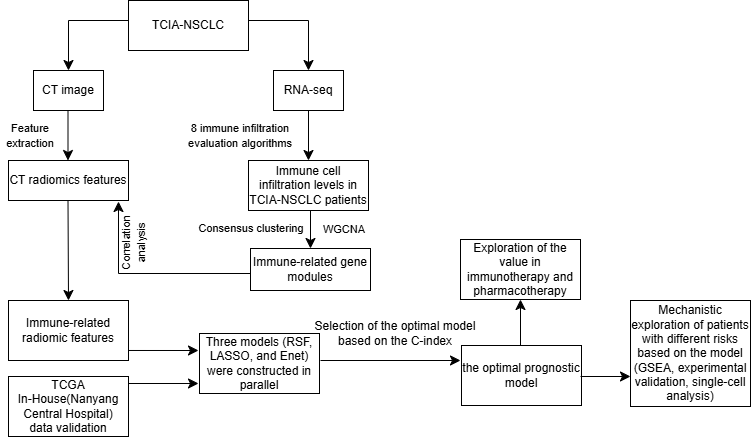

Supplement: Supplementary file 1 [file DataSheet1.zip › Supplementary_Material.docx]
