# Supplementary figures and images for: CT-based immune radiomic signature for prognosis and prediction of immunotherapy and anticancer drug response in NSCLC
Source: Front Immunol. 2026 Apr 29;17:1767389. doi: 10.3389/fimmu.2026.1767389 (PMC13168087; doi:10.3389/fimmu.2026.1767389)

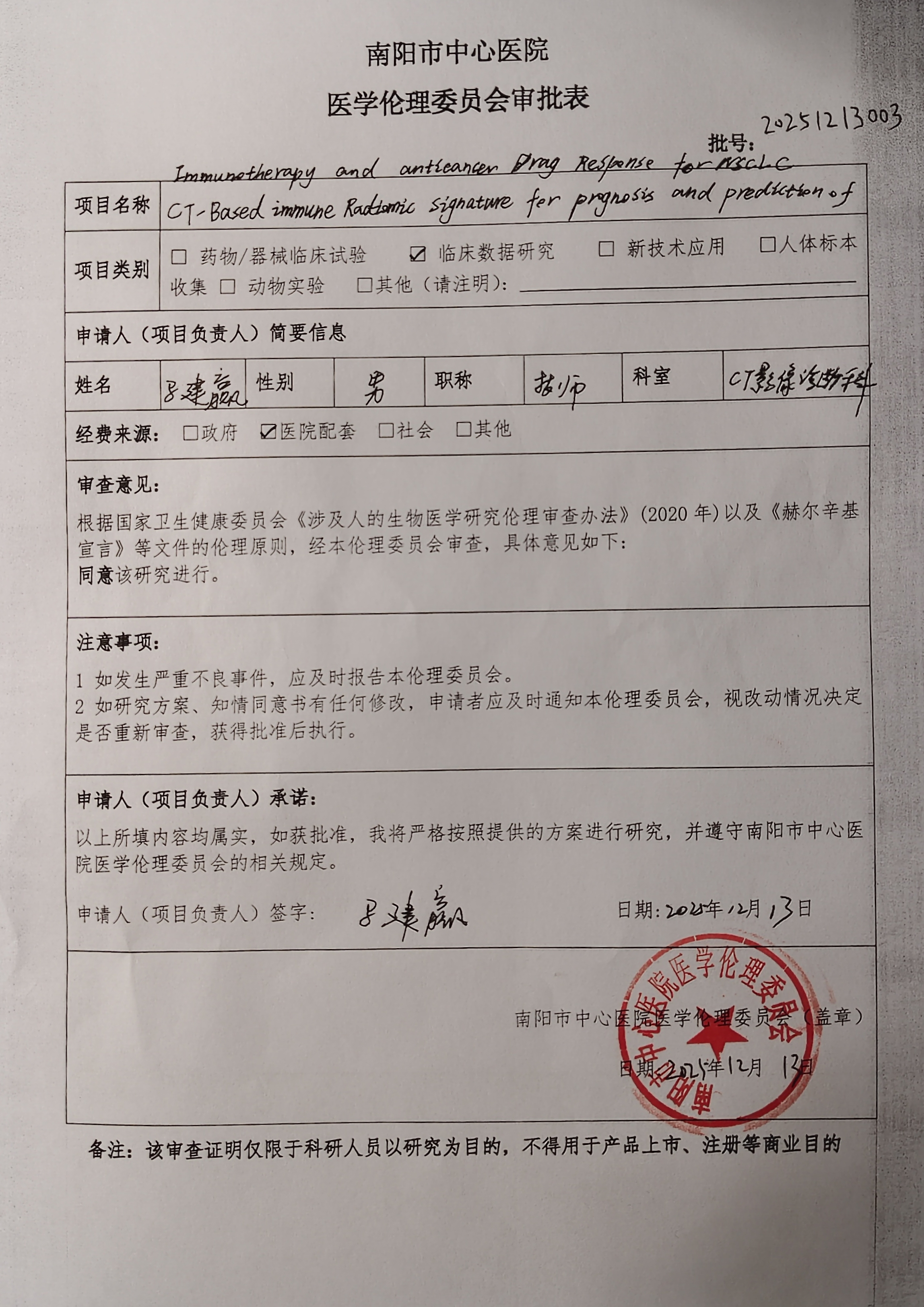

Supplement: Supplementary file 1 [file DataSheet1.zip › Ethics Approval.jpg]
